# The pharmacoepigenetic paradigm in cancer treatment

**DOI:** 10.3389/fphar.2024.1381168

**Published:** 2024-04-24

**Authors:** Belén Ocaña-Paredes, Sebastián Rivera-Orellana, David Ramírez-Sánchez, Juliana Montalvo-Guerrero, María Paula Freire, Samantha Espinoza-Ferrao, Adriana Altamirano-Colina, Paulina Echeverría-Espinoza, María José Ramos-Medina, Gabriela Echeverría-Garcés, Danilo Granda-Moncayo, Andrea Jácome-Alvarado, María Gabriela Andrade, Andrés López-Cortés

**Affiliations:** ^1^ Cancer Research Group (CRG), Faculty of Medicine, Universidad de Las Américas, Quito, Ecuador; ^2^ Facultad de Ingenierías y Ciencias Aplicadas, Universidad Internacional SEK, Quito, Ecuador; ^3^ German Cancer Research Center (DKFZ), Faculty of Biosciences, Heidelberg University, Heidelberg, Germany; ^4^ Centro de Referencia Nacional de Genómica, Secuenciación y Bioinformática, Instituto Nacional de Investigación en Salud Pública “Leopoldo Izquieta Pérez”, Quito, Ecuador; ^5^ Latin American Network for the Implementation and Validation of Clinical Pharmacogenomics Guidelines (RELIVAF-CYTED), Santiago, Chile; ^6^ Deutsche Gesellschaft für Internationale Zusammenarbeit Gmbh, Quito, Ecuador

**Keywords:** epigenetic drugs, histone deacetylase inhibitors, histone acetyltransferase inhibitors, histone methyltransferase inhibitors, DNA methyltransferase inhibitors, clinical trials

## Abstract

Epigenetic modifications, characterized by changes in gene expression without altering the DNA sequence, play a crucial role in the development and progression of cancer by significantly influencing gene activity and cellular function. This insight has led to the development of a novel class of therapeutic agents, known as epigenetic drugs. These drugs, including histone deacetylase inhibitors, histone acetyltransferase inhibitors, histone methyltransferase inhibitors, and DNA methyltransferase inhibitors, aim to modulate gene expression to curb cancer growth by uniquely altering the epigenetic landscape of cancer cells. Ongoing research and clinical trials are rigorously evaluating the efficacy of these drugs, particularly their ability to improve therapeutic outcomes when used in combination with other treatments. Such combination therapies may more effectively target cancer and potentially overcome the challenge of drug resistance, a significant hurdle in cancer therapy. Additionally, the importance of nutrition, inflammation control, and circadian rhythm regulation in modulating drug responses has been increasingly recognized, highlighting their role as critical modifiers of the epigenetic landscape and thereby influencing the effectiveness of pharmacological interventions and patient outcomes. Epigenetic drugs represent a paradigm shift in cancer treatment, offering targeted therapies that promise a more precise approach to treating a wide spectrum of tumors, potentially with fewer side effects compared to traditional chemotherapy. This progress marks a step towards more personalized and precise interventions, leveraging the unique epigenetic profiles of individual tumors to optimize treatment strategies.

## Introduction

### The history of epigenetics

The history of epigenetics traces back to ancient philosophical inquiries about organism development, with Aristotle proposing early theories about embryogenesis. The term “epigenesis” was later introduced around 1650 (Deichmann, 2016), following William Harvey’s 17th-century work, to describe the progressive development of an organism’s characteristics, challenging the prevailing idea of preformationism. This concept of epigenesis laid the foundation for modern epigenetics, sparking significant debates in the 19th century, particularly in the field of embryology about the role of chemical processes in trait expression (De la Peña and Vargas, 2018).

Conrad Waddington, often hailed as the father of epigenetics, further advanced the concept by defining epigenetics as the complex developmental process that links an organism’s genotype with its phenotype. This definition marked a pivotal shift towards the contemporary understanding of epigenetics (Bird, 2007; Deichmann, 2016).

Throughout the 20th century, the field of epigenetics expanded rapidly, especially with contributions from scientists like Arthur Riggs. Epigenetics came to be recognized as the study of heritable changes in gene expression that do not involve modifications to the DNA sequence itself (Riggs and Xiong, 2004; Felsenfeld, 2014). Significant breakthroughs, such as the identification of transcription markers like methylation and acetylation of histones by Vincent Allfrey and Alfred Misky, and further research linking epigenetic variations to diseases (e.g., cancer, systemic lupus erythematosus, and type II diabetes) by scientists like David Allis, underscored the field’s importance (Rakyan et al., 2011; Deichmann, 2016; Adams and Shao, 2022).

The advent of pharmacoepigenetics, heralded by the U.S. Food and Drug Administration (FDA)’s 2006 approval of azacytidine for treating myelodysplastic syndrome (Kaminskas et al., 2005), opened new avenues for therapeutic interventions. This approval was a significant milestone, establishing the relevance of epigenetic drugs in medical treatment. Currently, the field boasts thousands of drugs and compounds targeting epigenetic factors, with significant numbers receiving FDA approval for treating various conditions. Drugs such as decitabine, vorinostat, romidepsin, and panobinostat are notable examples, known for their roles in inhibiting methyltransferase and deacetylase, and are approved for the treatment of various diseases (Kringel et al., 2021).

The journey from Aristotle’s initial hypotheses to today’s advanced pharmacoepigenetic treatments highlights the remarkable evolution of epigenetic research (Claes et al., 2010). This dynamic progression has led to the development of sophisticated treatment strategies that consider the intricate interplay between genetics and environmental factors. The importance of nutrition, inflammation control, and circadian rhythm regulation in modulating drug responses and enhancing treatment efficacy has gained recognition in recent years (Masri et al., 2015; Nahmias and Androulakis, 2021; Barrero et al., 2022; Tan et al., 2022). These factors have been identified as crucial modifiers of the epigenetic landscape, directly impacting the effectiveness of pharmacological interventions and patient outcomes (Pérez-Villa et al., 2023). This progression underscores the transformative impact of epigenetics in understanding developmental biology and its potential to revolutionize therapeutic approaches, promising more personalized and effective treatment strategies (Felsenfeld, 2014).

## Gene expression and epigenetics

Eukaryotic organisms display complex genomic structures, distinctly different from those of prokaryotes, characterized by a well-defined nucleus, the formation of chromatin, and the critical role of histones (Koonin, 2010). This intricate architecture is essential for gene expression and chromosome regulation, either facilitated or restricted by DNA’s structural domains. Chromatin, existing as either tightly packed heterochromatin or less condensed euchromatin, plays a vital role in gene regulation and epigenetic processes. Heterochromatin, in particular, is crucial for organizing large chromosome domains, necessary for proper chromosome segregation (Grewal and Moazed, 2003). Its formation involves a detailed process of histone modifications by silencing complexes, emphasizing the importance of histone tails and the role of non-coding RNAs in the formation of epigenetic domains. The three-dimensional architecture of the genome, influenced by its function, indicates that chromatin’s structural features serve as modulators of genome activity. This dynamic, elucidated through various scientific approaches, highlights the development of structural chromatin features, the diversity and heterogeneity of nuclear architecture, and the evolutionarily conserved traits of genomes, such as plasticity and robustness (Misteli, 2020). Moreover, the complexity of eukaryotic gene regulation, as detailed by the central dogma of molecular biology and further complicated by processes like mRNA maturation, underscores the sophisticated nature of eukaryotic genetic regulation.

In eukaryotes, it is important to note that not all DNA is transcribed into RNA, and not all RNA is translated into proteins. The DNA includes non-coding sequences and gene-flanking regions, while RNA can regulate the expression of other genes (Hernández et al., 1995). The amount of mRNA transcribed can affect gene expression levels, which depend on both the integrity of DNA and the accessibility to transcriptional and translational machinery. DNA damage from mutagens can lead to mutations, while intact genes that are inaccessible to this machinery, or lack recognizable sections, may not be expressed. This variability is part of what is known as the epigenome (Corella and Ordovas, 2017), enabling cells with the same genome to exhibit different phenotypes within an organism.

Gene expression is also influenced by the gene’s location in euchromatin regions, where reduced compaction due to lower electromagnetic charges from DNA and histones allows easier access for transcriptional machinery. Nucleosomes further impact DNA accessibility by organizing the DNA-protein complex into a three-dimensional structure (Shi, 2007).

Epigenetic regulation, which involves modifying proteins and pathways without altering the DNA sequence, is affected by both the organism’s environment and its cellular microenvironment (Bedregal et al., 2010). This regulation includes mechanisms such as gene silencing and activation, and the control of gene promoters and repressors, making epigenetic changes heritable and reversible. Non-Mendelian inheritances, such as prions and non-coding RNAs, contribute to phenotypic expression through epimutations, which mainly alter the three-dimensional conformation of chromatin (Shi, 2007).

Epimutations can change DNA or chromatin charges, or the structure of histone proteins, affecting gene expression. The proximity of a gene to the cellular periphery can also influence its activation (Shi, 2007). These complex changes represent alterations in entire regulatory mechanisms, not just structural components. The epigenetic state of a cell is constantly modified, allowing for optimal function in diverse environments (Horsthemke, 2006). Lastly, epigenetic alterations can disrupt cellular pathways, potentially leading to cancer.

## Molecular underpinnings of epigenetic modifications that lead to cancer

Epigenetic regulation encompasses several molecular mechanisms that operate at different biological scales, notably the post-translational modifications of histones, which are central to the regulation of gene expression (Table 1) (Mohtat and Susztak, 2010; Bell and Spector, 2011). These modifications include, but are not limited to, acetylation, methylation, ubiquitination, and phosphorylation, each carrying distinct regulatory functions (Cao et al., 2005; Glozak et al., 2005; Cavagnari, 2012). DNA-level modifications, such as DNA methylation and ATP-dependent chromatin remodeling, also play pivotal roles in altering chromatin architecture, thereby influencing gene accessibility and expression. Additionally, non-coding RNAs, telomere positioning, genomic imprinting, polycomb and trithorax group proteins, prion-like factors, X-chromosome inactivation, and sumoylation processes contribute to the complex landscape of epigenetic regulation (Blackburn, 2001; Shi, 2007; Bracken and Helin, 2009; Cotton et al., 2015).

**TABLE 1:** Landscape of epigenetic mechanisms involved in cancer.

Mechanism	Description	Cancer types	Reference
DNA methylation	Addition of methyl groups to DNA, leading to gene silencing	Colorectal, breast, lung	Jones and Baylin (2007)
Histone acetylation	Addition of acetyl groups to histones, usually associated with gene activation	Leukemia, glioblastoma	Glozak et al. (2005)
Histone methylation	Methylation of histones, affecting gene expression depending on the side and state of methylation	Prostate, lymphoma	Greer and Shi (2012)
Non-coding RNAs	RNA molecules that regulate gene expression, including microRNAs and long non-coding RNA	Liver, breast, pancreas	Esteller (2011)
Chromatin remodeling	Reorganization of chromatin, affecting DNA accessibility and gene expression	Melanoma, sarcoma	Wilson and Roberts (2011)
RNA interference	Small RNA molecules inhibit gene expression or translation, impacting cancer genes	Ovarian, kidney	Bader (2012)
Histone phosphorylation	Addition of phosphate groups to histones, involved in DNA repair and chromosome condensation	Breast, colorectal	Rossetto et al. (2012)
Histone ubiquitination	Addition of ubiquitin to histones, involved in DNA repair and gene expression regulation	Colorectal, prostate	Cao et al. (2005)
DNA hydroxymethylation	Conversion of methylated DNA to hydroxymethylated DNA, associated with gene activation	Melanoma, gliomas, liver	Lian et al. (2012)
Polycomb repression	Polycomb group proteins repress gene expression through histone modifications	Breast, endometrial	Bracken and Helin (2009)
Nucleosome positioning	Arrangement of nucleosomes along DNA, influencing gene expression by accessibility	Leukemia, melanoma	Voigt et al. (2013)
Histone variants	Incorporation of histone variants into nucleosomes, affecting chromatin structure and gene expression	Glioblastoma, sarcoma	Talbert and Henikoff (2010)
Telomere positioning	Influence of telomere structure and location on gene expression	Lung, bladder	Blackburn (2001)
Sumoylation	Addition of SUMO proteins to target proteins, influencing stability and activity	Breast, thyroid	Seeler and Dejean (2003)
Enhancer RNAs	Non-coding RNAs transcribed from enhancer regions that regulate gene expression	Prostate, colorectal	Li et al. (2013)
DNA demethylation	Removal of methyl groups from DNA, often leading to gene activation	Colorectal, gastric	Wu and Zhang (2010)
Chromatin accessibility	Degree to which DNA is exposed and accessible to transcription factors	Lymphoma, leukemia	Thurman et al. (2012)
X-chromosome inactivation	Inactivation of one of the X chromosomes in females	Breast, ovarian	Cotton et al. (2015)
Genomic imprinting	Parent-specific gene expression due to epigenetic marks	Ovarian, testicular	Ferguson-Smith (2011)

Histone PTMs are dynamic and multifaceted, capable of occurring concurrently and influencing the interactions between histones and DNA. Such modifications can lead to chromatin compaction and gene silencing or chromatin relaxation, enhancing transcriptional activity (Zhao and Shilatifard, 2019). The specificity of these modifications, including their type and location, underlines the intricate control of gene expression and phenotypic outcomes (Cavagnari, 2012).

The interplay between histone modifications and chromatin structure is further complicated by cis and trans effects, with cis effects modifying chromatin at the level of internucleosomal contacts, and trans effects arising from the association of various proteins with the chromatin (Esteller, 2007; Talbert and Henikoff, 2010; Cavagnari, 2012; Greer and Shi, 2012). This complexity is augmented by the enzymatic systems responsible for adding or removing histone PTMs, which also target non-histone proteins, thereby extending the scope of epigenetic regulation (Shi, 2007; Voigt et al., 2013).

Among the myriad of histone PTMs, acetylation and methylation are particularly noteworthy. Acetylation, typically associated with transcriptional activation, is mediated by histone acetyltransferases (HATs) and reversed by histone deacetylases (HDACs) (Glozak et al., 2005; Shi, 2007; Gujral et al., 2020). Methylation, however, exhibits a dual role in gene regulation, dependent on the specific amino acids modified and their methylation state, adding layers to the regulatory network (Zhang et al., 2023).

Histone phosphorylation and ubiquitination are additional modifications with significant roles in gene expression and chromatin dynamics. Phosphorylation, often linked to chromatin condensation, is mediated by specific kinases (Shi, 2007; Rossetto et al., 2012), whereas co-activator-mediated ubiquitination is definitively associated with gene activation (Talukdar and Chatterji, 2023). Other modifications, such as glycosylation and sumoylation, although less understood, are recognized for their contribution to chromatin structure and function (Seeler and Dejean, 2003; Ramírez et al., 2009).

DNA methylation, a key epigenetic mark, occurs predominantly at CpG sites and plays a critical role in gene silencing and chromatin remodeling (Jones and Baylin, 2007; Zhang et al., 2023). The intricate balance between methylation, demethylation, hydroxymethylation is crucial for cellular function and is tightly regulated by DNA methyltransferases and other chromatin-associated proteins (Wu and Zhang, 2010; Lian et al., 2012; Dhar et al., 2021).

In the context of cancer epigenetics, this field has made significant strides in understanding how DNA methylation and histone modifications contribute to tumorigenesis (Baylin and Jones, 2016). This review highlights the importance of hypermethylation in silencing tumor suppressor genes, CpG islands, and individual genes, which has implications for the early detection and classification of cancer. Tumor suppressor proteins such as INK4A, INK4B, and APC, along with enzymes and cell adhesion proteins like GSTP1, MGMT, and CDH1, have been studied extensively in cancers of the prostate, liver, stomach, colon, and breast. These studies reveal tissue-specific methylation patterns, underscoring the complexity of epigenetic regulation in different cell types (Esteller, 2002; Gonzalo et al., 2008; Bouyahya et al., 2022).

Microsatellite instability, another focus of cancer epigenetics research, is closely linked to colorectal cancer and Lynch syndrome (Gusev, 2019). It arises from length changes in microsatellites, increasing susceptibility to replication errors and leading to genetic alterations that drive cancer progression (Toledo González and Cruz-Bustillo Clarens, 2005).

To identify methylated sites within the genome, researchers employ a variety of techniques, including methylation-sensitive enzymes, chemical conversion of methylated cytosines using sodium bisulfite, and immunological methods that capture DNA-methyl cytosine complexes (Frommer et al., 1992). These approaches are pivotal in mapping the epigenetic landscape of cancer cells (Weber et al., 2005).

Epigenetic therapies, particularly those targeting DNA methylation like azacitidine, aim to reactivate silenced genes, offering new strategies for cancer treatment. These drugs modify the epigenetic landscape, potentially reversing the gene silencing that contributes to cancer development (Issa and Kantarjian, 2009; Gailhouste et al., 2018).

Chromatin remodeling, a key aspect of epigenetic regulation, involves both covalent histone modifications and ATP hydrolysis mechanisms (Zhou et al., 2016). This remodeling facilitates access to transcriptional machinery by displacing nucleosomes, leading to nucleosomal sliding or eviction and consequent changes in gene expression. The SWI/SNF complex is notable for its role in these processes, highlighting the dynamic interplay between chromatin structure and gene regulation (Schwabish and Struhl, 2007; Wilson and Roberts, 2011; Cavagnari, 2012).

RNA molecules, smaller than mRNA, play crucial roles in regulating gene expression by managing DNA exposure for transcription and translation. The regulation of gene expression by microRNAs (miRNAs), small interfering RNAs (siRNAs), enhancer RNAs (eRNAs), and other non-coding RNAs is vital for cellular processes like differentiation, immune response, and cell proliferation (Bader, 2012; Li et al., 2013; Lam et al., 2015). These RNAs contribute to cancer development and progression by altering the transcriptome, offering potential targets for therapeutic intervention (Esteller, 2011; Gusev, 2019).

In summary, the study of cancer epigenetics encompasses the investigation of DNA methylation patterns, histone modifications, chromatin remodeling, and RNA-mediated gene regulation. These components interplay to control gene expression, with aberrations in these processes contributing to the onset and progression of cancer. Understanding these mechanisms provides valuable insights into cancer biology and opens avenues for novel diagnostic and therapeutic strategies.

## Immune system and epigenetics

The immune system in vertebrates is a complex and finely tuned entity, governed not only by signaling pathways but also by a network of epigenetic mechanisms (Busslinger and Tarakhovsky, 2014). These epigenetic regulations are essential for the immune system’s ability to defend against various pathogens, illustrating the system’s sensitivity to both internal and external stimuli. The activity of immune cells, such as macrophages, and their components like toll-like receptors (TLRs), is subject to precise epigenetic control (Fitzgerald and Kagan, 2020). For example, the regulation of inflammatory cytokine genes in macrophages is mediated by histone modifications, particularly the induction of histone lysine acetylation, which is crucial for cytokine gene transcription (Obata et al., 2015).

Bromodomain and extraterminal (BET) proteins, which bind to acetylated lysines, play a significant role in modulating cytokine gene expression (Obata et al., 2015). Studies have shown that synthetic compounds like I-BET can inhibit the expression of inflammatory cytokines by disrupting the interaction between BET proteins and acetylated histones, thereby suppressing the immune response to lipopolysaccharides in macrophages (Nicodeme et al., 2010; Guo et al., 2019; Wang et al., 2021).

Histone deacetylase enzymes, specifically class IV HDAC, are also pivotal in the immune response, targeting histones near the interleukin (IL) 10 gene to compact chromatin and inhibit transcription. This suppression of IL-10, an anti-inflammatory cytokine, highlights the role of epigenetic regulation in modulating immune responses (Villagra et al., 2009).

Methyltransferases further influence the immune landscape, particularly in macrophages. Ash1l, an enzyme that methylates lysine 4 on histone H3 (H3K4), enhances the expression of the A20 gene (Xia et al., 2013), which plays a role in modulating inflammatory responses by inhibiting certain signaling pathways. This methylation leads to the suppression of NF-kB and MAPK signaling pathways, reducing the production of IL-6, an inflammatory cytokine (Xia et al., 2013).

Another H3K4 methyltransferase, Wpp7, is crucial for the antimicrobial response in macrophages (Li et al., 2022). The absence of Wpp7 leads to reduced expression of glycosylphosphatidylinositol (GPI)-anchored proteins, affecting the macrophages’ response to microbial stimuli due to the lack of CD14, a GPI-anchored protein necessary for effective microbial recognition (Austenaa et al., 2012).

This intricate network of epigenetic regulation, from histone modifications to the activity of specific enzymes, underscores the delicate balance maintained in the immune system. Disruptions in this balance can lead to immunological disorders, such as inflammation, demonstrating the critical role of epigenetic controls in immune regulation (Xu et al., 2023).

## Before cancer treatment

### The role of epigenetic signatures in the pharmacoepigenetic treatments of cancer

Cancer is characterized by complex genomic and epigenomic alterations that drive its development and progression. Among these, epigenomic changes, including DNA methylation, histone modifications, and the reprogramming of non-coding RNAs, play a pivotal role due to their reversible nature, making them attractive targets for therapeutic intervention (Lu et al., 2020). DNA methylation, the most extensively studied epigenetic modification, often occurs early in cancer and influences gene expression by altering the methylation status of oncogenes or tumor suppressor genes (Diaz-Lagares et al., 2016). This can lead to the dysregulation of non-coding RNAs, further impacting the regulation of mRNA targets and potentially contributing to oncogenesis (Nishiyama and Nakanishi, 2021; Tonmoy et al., 2022).

Histone modifications encompass a wide range of changes, such as acetylation, methylation, and phosphorylation, which affect chromatin structure and subsequently the accessibility of transcription factors to DNA (Kouzarides, 2007). These modifications not only regulate gene expression directly but also influence the activity of non-coding RNAs involved in post-transcriptional gene regulation (Ferro et al., 2017).

The concept of the cancer epigenome encompasses the global aberrant epigenetic marks found across various tumor types, a phenomenon known as epigenetic instability. This hallmark of cancer is integral to developing diagnostic and prognostic epigenetic signatures, as seen in the hypermethylation of genes in prostate cancer, altered CpG island methylation in breast cancer associated with poor outcomes, and the silencing of tumor suppressor genes like CDO1 across multiple cancer types due to DNA methylation (Brait et al., 2012; Batra et al., 2021).

Specific epigenetic patterns, such as DNA hypermethylation in lung adenocarcinoma related to smoking history and changes in histone modification levels in colorectal cancer (Gezer et al., 2015; Bakulski et al., 2019), highlight the heterogeneity and complexity of cancer epigenetics (Brait et al., 2012; Diaz-Lagares et al., 2016). These findings underpin the development of diagnostic tools and pharmaco-epigenetic therapies, exemplified by FDA-approved colorectal cancer screening tests that assess DNA methylation (Koch et al., 2018; Batra et al., 2021).

However, the high heterogeneity among tumors, coupled with environmental influences like smoking and aging-related epigenetic changes, presents significant challenges in identifying cancer-specific epigenetic landscapes and therapeutic targets. Distinguishing cancer-related epigenetic modifications from those associated with aging is crucial for advancing precision medicine and optimizing pharmaco-epigenetic approaches to cancer treatment. This differentiation is essential in developing targeted therapies and enhancing the efficacy of epigenetic-based interventions in cancer care (Pérez et al., 2021).

## During cancer treatment

### Histone deacetylase inhibitors

**FIGURE 1:**
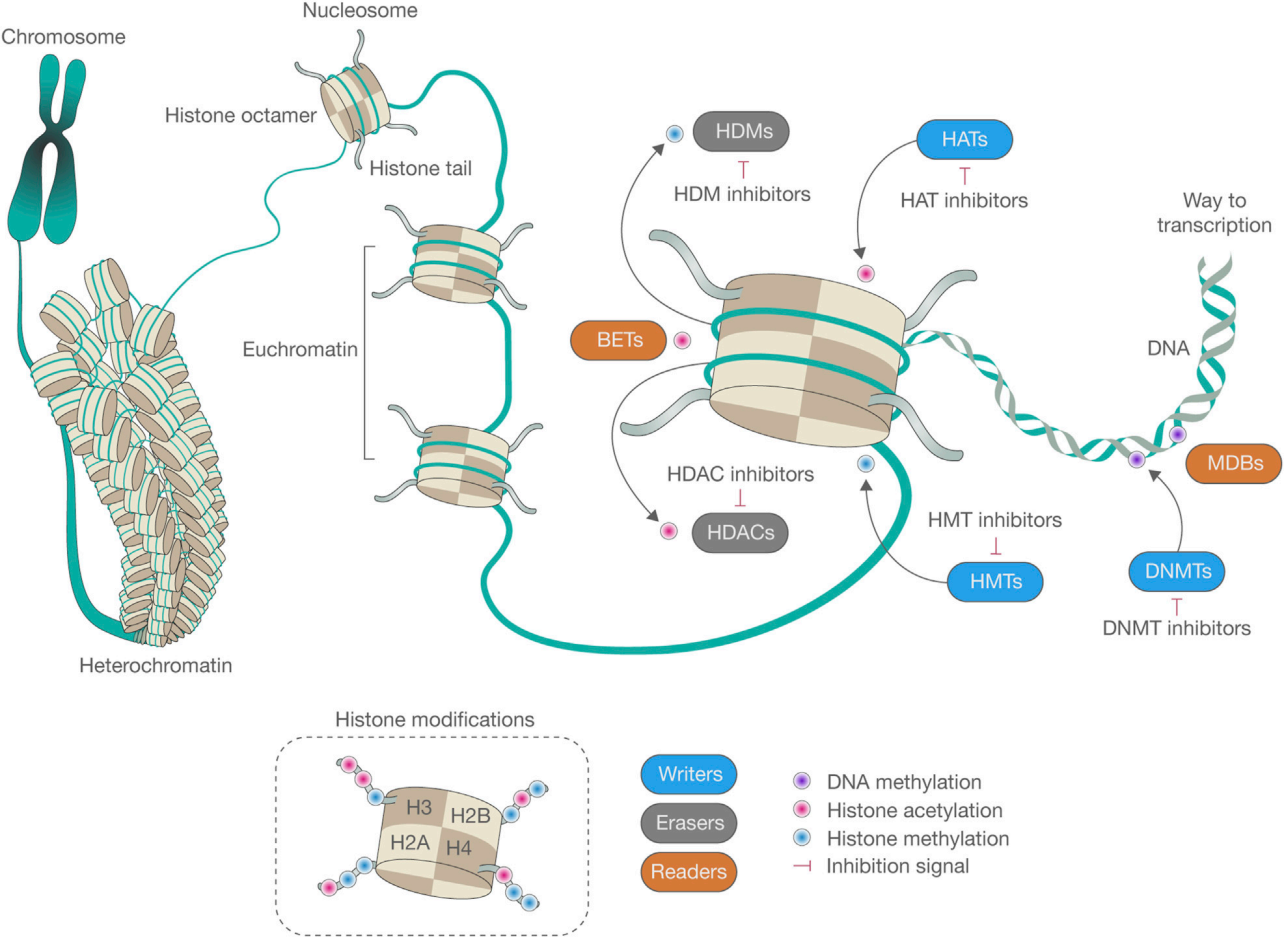
The epigenetic machinery plays an essential role in shaping the conformation of chromatin and regulating genome functionality. DNA is intricately packed and wound around a core composed of histone octamers, thus forming nucleosomes, the fundamental structural units of chromatin. This sophisticated network of epigenetic modifications, encompassing DNA methylation and histone modifications, profoundly impacts the structure of chromatin and the functionality of the genome. Central to epigenetics are enzymes that serve three primary roles: adding (writers), recognizing (readers), and removing (erasers) epigenetic marks on DNA or histone tails. DNA methylation, primarily carried out by DNA methyltransferases (DNMTs), can be reversed by ten-eleven translocation enzymes (TETs) or can diminish progressively over successive cell divisions. Among histone modifications, acetylation and methylation have been extensively studied. The equilibrium of histone methylation is controlled by the opposing activities of histone methyltransferases (HMTs) and histone demethylases (HDMs). In a similar vein, histone acetylation levels are modulated by the concerted efforts of histone acetyltransferases (HATs) and histone deacetylases (HDACs), which add or remove acetyl groups from lysine residues on the histone tails, respectively. This “epigenetic code” is interpreted by specific reader or effector proteins that selectively bind to certain types of modifications. For instance, methyl-CpG-binding domain (MBDs) proteins bind to methylated DNA, whereas bromodomain and extraterminal domain proteins (BETs) recognize acetylated lysines. These epigenetic modifications play a critical role in altering chromatin conformation, leading to either the transcriptional silencing or activation of genes, often through the recruitment of additional proteins to these sites.

Histone deacetylase inhibitors are essential chemotherapeutic agents used in cancer therapy, serving as both cytotoxic and cytostatic drugs (Ferrarelli, 2016; Eckschlager et al., 2017) (Figure 1). The FDA has approved HDAC inhibitors such as vorinostat, romidepsin, belinostat, and panobinostat (Table 2). Additionally, HDAC inhibitors like chidamine, valproic acid, entinostat, tacedinaline, quisinostat, and resminostat are currently under investigation (Mann et al., 2007; Piekarz et al., 2009; San-Miguel et al., 2014; O’Connor et al., 2015; Mendez et al., 2019; Ochoa et al., 2023). These inhibitors function by blocking the removal of acetyl groups from histones, thereby preserving an open chromatin structure essential for enhancing gene expression (Gonzalo et al., 2008; Stimson et al., 2009). Such an open chromatin structure is critical for inhibiting tumor cell activity and repressing gene expression involved in processes like angiogenesis, cell cycle disruption, immunity, and cell survival (Gonzalo et al., 2008).

**TABLE 2:** FDA-approved epigenetic inhibitors.

Inhibitor	Drug	First approval	Target	Cancer treatment	Relevant clinical trial	Reference
HDAC	Vorinostat	2006	HDAC 1, 2, 3, and 6	Cutaneous T-cell lymphoma	NCT00091559	Mann et al. (2007)
HDAC	Romidepsin	2009	HDAC 1, 2, 4, and 6	Cutaneous T-cell lymphoma, peripheral T-cell lymphoma	NCT00106431	Piekarz et al. (2009)
HDAC	Belinostat	2014	HDAC 1, 2, 3, and 10	Peripheral T-cell lymphoma	NCT00106431	O’Connor et al. (2015)
HDAC	Panobinostat	2015	HDAC 1, 2, 3, 6, and 10	Multiple myeloma (in combination with bortezomib and dexamethasone)	NCT01023308	San-Miguel et al. (2014)
HMT	Tazemetostat	2020	EZH2	Epitheloid sarcoma, follicular lymphoma	NCT01897571	Italiano et al. (2018)
DNMT	Azacitidine	2004	DNMT	Myelodysplastic syndromes, acute myeloid leukemia, chronic myelomonocytic leukemia	NCT00071799	Fenaux et al. (2010)
DNMT	Decitabine	2006	DNMT	Myelodysplastic syndromes, acute myeloid leukemia	NCT00313586	Kantarjian et al. (2006)

As epigenetic modulators, HDAC inhibitors alter the expression of histone and non-histone proteins without changing the DNA sequence itself. They help restore normal cell differentiation and apoptotic functions by maintaining histone acetylation, modifying chromatin structure, and facilitating access for transcription factors (Bolden et al., 2006; Mayr et al., 2021).

Histone acetylation, promoted by HAT/KAT enzymes, leads to changes in gene expression and chromatin structure. Conversely, HDAC enzymes act as transcriptional repressors by removing these acetyl groups. Such histone deacetylation results in transcriptional silencing, either through increased charge density in histone N-terminal groups or chromatin compaction, thus reducing transcription accessibility (Gallinari et al., 2007; Seto and Yoshida, 2014).

HDACs are targeted by several classes of inhibitors, including hydroxamic acids, cyclic peptides, aliphatic fatty acids, benzamides, epoxy ketones, and hybrids, categorized by their chemical structures and action mechanisms (Hess-Stumpp et al., 2007; Jain and Zain, 2011; Ghasemi, 2020; Lu et al., 2020). These inhibitors, affecting the 18 known HDACs through zinc-dependent or NAD + -dependent mechanisms, offer therapeutic potential by inducing apoptosis, cell cycle arrest, and modifying non-coding RNA expression (Bose et al., 2014; Suraweera et al., 2018). They boost the acetylation of genes that regulate the cell cycle and apoptosis, like p53, thereby inducing apoptosis through various pathways (Kulka et al., 2020).

Nonetheless, the clinical application of HDAC inhibitors encounters challenges such as drug resistance, often due to the overexpression of proteins like CDC25A in tumor cells, and side effects like nausea, vomiting, headache, and fluid and electrolyte imbalances (Liu et al., 2020; Wang et al., 2023). Addressing these issues requires patients to adhere to medical advice specifically tailored to their treatment plan. Comprehending the molecular mechanisms of HDAC inhibitors, including their effects on chromatin structure and gene expression, is vital for refining cancer treatment strategies and overcoming resistance and adverse effects (Matore et al., 2022).

### Histone acetyltransferase inhibitors

Histone acetyltransferases play a crucial role in regulating gene expression by acetylating histones, thereby making DNA more accessible for transcription (Sterner and Berger, 2000). Instead of inhibiting, they enhance the acetyltransferase activity on lysine residues of histones, a process essential for transcriptional activation associated with euchromatin. This activity is vital for suppressing tumor cell growth and inhibiting cellular mitosis (Dalvoy Vasudevarao et al., 2012; Sabnis, 2021). However, developing inhibitors that target HATs has been challenging, with most potential inhibitors still in the experimental or preclinical stages.

HAT inhibitors aim to target specific human enzymes such as CREBBP, linked to acute myeloid leukemia, as well as CDY1 and CDY2, important for erythropoiesis and spermatogenesis, and CLOCK, crucial for regulating circadian rhythms (Mullighan et al., 2011; Pérez-Villa et al., 2023). The significance of HAT inhibitors spans various enzymes implicated in cancers, including hematological neoplasms like diffuse large B-cell lymphoma, non-Hodgkin lymphoma, acute lymphocytic leukemia, and solid tumors such as colorectal, breast, and prostate cancer (Wu et al., 2020). Noteworthy inhibitors under investigation include anacardic acid, C646, curcumin, garnicol, and MB-3.

The exploration of HAT inhibitors has raised concerns about potential side effects, which might include issues typical of chemotherapeutic agents, such as cytotoxicity and off-target effects (Manzo et al., 2009). Concerns about chemotherapy resistance, potentially driven by the activation of multidrug resistance genes and increased growth factors, also underscore the need for careful therapeutic design and monitoring.

HATs are bisubstrate enzymes that catalyze the transfer of an acetyl group from the cofactor acetyl coenzyme A (Ac-CoA) to a lysine substrate on histones, playing a pivotal role in gene regulation. They function through mechanisms including a random ternary complex, an obligatory ordered ternary complex requiring a general base like glutamic acid, and a Ping-Pong mechanism, wherein Ac-CoA binds first, transferring the acetyl group to an enzyme’s amino acid before CoA binds to the substrate (Wapenaar and Dekker, 2016).

Research continues to focus on developing small molecule HAT inhibitors as potential therapeutic agents. Efforts include designing HAT mimics, utilizing natural products, and conducting virtual screenings to identify high-performance HAT inhibitors. Examples such as KAT2B, KAT3B, KAT5, and KAT5 ESA1 are promising due to their selectivity but face challenges including low metabolic stability and lack of cellular permeability (Poziello et al., 2021). Natural product-based HAT inhibitors, such as garcinol, show potential in preclinical models for inhibiting cancer cell growth, highlighting the ongoing challenge of balancing the biological activity of HAT inhibitors with their drug applicability for treating diseases like cancer (Kopytko et al., 2021).

### Histone methyltransferase inhibitors

Histone methyltransferase (HMT) inhibitors are emerging as significant agents in cancer therapy, targeting enzymes responsible for methylating lysine or arginine residues on histones, especially on proteins H3 and H4 (Figure 1). This methylation plays a critical role in epigenetic regulation of gene expression, including silencing tumor suppressor genes in cancer cells. By inhibiting HMTs, these drugs aim to correct aberrant methylation patterns, potentially reactivating genes that can suppress tumor growth and affect processes such as cell replication, differentiation, apoptosis, angiogenesis, and senescence (Kim and Bae, 2011).

Understanding chromatin structure is fundamental. Every cell in the human body contains identical DNA, packaged with histones into chromatin. The nucleosome, comprising an octameric core of histones (two copies each of H2A, H2B, H3, and H4) wrapped around a segment of DNA 145–147 base pairs in length, forms the basic unit of chromatin (Simon and Lange, 2008).

Histones regulate gene expression through various epigenetic mechanisms, including methylation, which occurs on lysine and arginine residues and involves enzymes such as G9a. G9a, a histone methyltransferase, is responsible for the monomethylation and dimethylation of histone H3 lysine 9 (H3K9), influencing chromatin structure and gene expression (Robertson, 2001; Liu et al., 2015). Methylation, particularly by G9a, plays a part in coordinating gene regulation along with DNA methyltransferases and demethylases.

Tazemetostat (an EZH2 inhibitor) has received FDA approval for treating epithelioid sarcoma and certain types of follicular lymphoma, marking a significant achievement in the clinical use of HMT inhibitors (Table 2) (Italiano et al., 2018). Other inhibitors in development include GSK2816126 for EZH2, pinometostat for DOT1L, GSK3326595 and JNJ-64619178 for PRMTs, and GSK2879552 and iadademstat for LSD1. Tazemetostat specifically targets EZH2, which is implicated in suppressing tumor genes and epithelial-mesenchymal transition (EMT)-related genes such as p16INK4a and e-cadherin (Baell et al., 2018).

However, HMT inhibitors can cause side effects, including nausea and vomiting, with some patients experiencing symptoms akin to tumor lysis syndrome. Therefore, patients often require close monitoring, especially during initial treatment cycles. Resistance to HMT inhibitors can arise from mutations in the tumor’s genomic sequence, emphasizing the need to understand the molecular mechanisms of action to optimize cancer treatment and manage resistance (Wang et al., 2023).

Histones undergo modifications such as acetylation and methylation. Acetylation neutralizes the positive charge on lysine residues, reducing histone-DNA interactions and leading to more relaxed chromatin conducive to gene expression (Liu et al., 2015). The effect of methylation on chromatin and gene expression depends on the specific residue methylated. These epigenetic modifications are crucial targets for drug development, as demonstrated by the approval of DNA methyltransferase and histone deacetylase inhibitors (Pócza et al., 2016).

In the epigenetic landscape, enzymes like G9a are termed “writers” for adding chemical groups, “readers” recognize these modifications, and “erasers” remove them (Subramaniam et al., 2014). Targeting G9a and other HMTs is a promising strategy for cancer therapy, underscoring the significance of these “writers” in maintaining malignant phenotypes and regulating gene expression (Ghoshal and Bai, 2007; Morera et al., 2016).

### DNA methyltransferase inhibitors

DNA methyltransferase (DNMT) inhibitors are increasingly recognized as essential agents in cancer therapy, specifically targeting enzymes responsible for methylating cytosine residues within CpG dinucleotides in DNA (Jin and Robertson, 2013). This methylation is fundamental to the epigenetic regulation of gene expression, including the silencing of tumor suppressor genes in cancer cells. By inhibiting DNMTs, these drugs aim to correct aberrant methylation patterns, potentially leading to the reactivation of genes that can suppress tumor growth and impact key processes such as cell replication, differentiation, apoptosis, angiogenesis, and senescence (Kim and Bae, 2011).

Histones play a critical role in regulating gene expression through various epigenetic mechanisms, notably methylation at cytosine residues, primarily carried out by DNMT enzymes such as DNMT1, DNMT3A, and DNMT3B (Apuri and Sokol, 2016). This methylation usually leads to gene silencing, playing a key role in coordinating gene regulation alongside DNA methyltransferases and demethylases.

FDA-approved DNMT inhibitors, including azacitidine and decitabine, are designated for treating myelodysplastic syndromes, acute myeloid leukemia, and chronic myelomonocytic leukemia (Table 2) (Kantarjian et al., 2006; Fenaux et al., 2010). Functioning as nucleoside analogs, these inhibitors integrate into DNA, leading to DNA hypomethylation (Giri and Aittokallio, 2019). At therapeutic doses, they primarily exhibit a cytostatic effect, enabling the reactivation of previously silenced genes (Traynor et al., 2023). Additionally, compounds like guadecitabine and CC-486 (oral azacitidine) are being evaluated in clinical trials for various conditions but have not yet been approved by the FDA (Ochoa et al., 2023).

However, repeated use of DNMT inhibitors can result in drug resistance, characterized by mutations and gene overexpression that enhance tumor cell survival against treatment (Laranjeira et al., 2023). DNMT inhibitors can cause side effects such as nausea, vomiting, dehydration, headache, anorexia, and myelosuppression (anemia, neutropenia, thrombocytopenia), necessitating close monitoring, particularly during the initial treatment cycles (Chen et al., 2023).

Current research focuses on developing non-nucleoside DNMT inhibitors with minimal off-target effects and enhanced specificity. Experimental compounds, including non-nucleoside analogs, are under investigation for their ability to more selectively block DNMT activity and reactivate tumor suppressor genes (Traynor et al., 2023).

In cancer treatment, DNMT inhibitors have demonstrated potential across various cancers, including bladder and gastric cancer, by inducing the overexpression of tumor suppressor genes, inhibiting tumor cell growth, and promoting apoptosis (Norollahi et al., 2019; Juárez-Mercado et al., 2020). These outcomes underscore the significant potential of DNMT inhibitors in clinical trials and experimental models. The DNMTs, as a conserved family of cytosine methyltransferases, are crucial for epigenetic regulation. Targeting these enzymes offers a strategic approach to developing cancer therapies, aiming to integrate such treatments into comprehensive cancer management strategies. As the field evolves, continued research on DNMT inhibitors is critical for advancing cancer treatment strategies.

### Combinational therapy

Combinational therapy marks a significant evolution in medical treatments by employing two or more therapeutic agents together, an approach that has been shown to be more effective than using a single agent alone (Bayat Mokhtari et al., 2017). This strategy is particularly beneficial in targeting proliferating cells, a common characteristic of cancer, but it also poses the risk of harming healthy cells alongside cancerous ones. Historically, combinational therapies have been instrumental in managing diseases with a high social impact, such as cardiovascular, metabolic, infectious, and autoimmune disorders. A prime example of this is the POMP regimen, introduced in 1965 for acute leukemia, which includes methotrexate, 6-mercaptopurine, vincristine, and prednisone, and has significantly improved remission rates in pediatric patients (Bayat Mokhtari et al., 2017).

In the context of cancer, combinational therapy often involves pairing standard chemotherapy or radiotherapy with a variety of drugs to address the complex nature of tumors, their compensatory mechanisms, and their rapid progression. This multi-targeted approach, through the sequential use of therapeutic agents, aims to reduce tumor growth, limit metastasis, and promote cell death in mitotically active cells, thus minimizing the risk of drug resistance (Ascierto and Marincola, 2011).

A newer strategy, restrictive combinations, is under clinical investigation. It focuses on optimizing drug dosing and scheduling to protect healthy cells while targeting cancer cells more effectively. For example, low doses of doxorubicin can induce a cell cycle arrest in healthy cells, shielding them from the cytotoxic effects of subsequent treatments like Taxol, which specifically targets cancer cells for mitotic arrest (Blagosklonny, 2008). The goal is to enhance the therapeutic impact by adding drugs in a manner that synergistically boosts their effectiveness (Bayat Mokhtari et al., 2017).

Drug repurposing is another innovative approach, where medications initially intended for other conditions are used in cancer treatment (Mokhtari et al., 2013). Acetazolamide, traditionally used for glaucoma, epilepsy, and altitude sickness, is one such drug being explored for cancer therapy. This is based on the observation that cancer cells frequently have high levels of carbonic anhydrase activity, contributing to their malignant properties. By inhibiting this enzyme, it's hoped that anticancer benefits can be achieved (Islam et al., 2016). Repurposing offers the advantages of using drugs with established safety profiles and potentially reducing treatment costs, making it a promising avenue in cancer therapy.

## After cancer treatment

### Epigenetic biomarkers in post-treatment evaluation

In the evolving landscape of cancer treatment and post-treatment evaluation, the role of epigenetic biomarkers is gaining prominence. The use of these biomarkers provides a practical method for assessing treatment efficacy and identifying potential risks that might emerge after treatment. This review explores the advancements and significance of various epigenetic biomarkers in different cancer types.

Among these biomarkers, DOK7, known as downstream of kinase, plays a pivotal role in the growth, migration, and invasion of cancer cells (Heyn et al., 2013). Intensive research has been conducted to understand its involvement in signaling pathways such as PI3K, PTEN, AKT. Clinical trials have shed light on how the overexpression of DOK7 can inhibit the activation of p-AKT and amplify the expression of PTEN, crucial for suppressing tumors associated with its overexpression. Specifically, in the context of breast cancer control, DOK7 is proposed as a powerful biomarker for forecasting the future presence of cancer and gauging its remission well in advance (Yue et al., 2021).

The field of bladder cancer detection also illustrates the potential of biomarkers like GDF15, TMEFF2, and VIM. These have shown a notable capability to detect the cellular absence of cancer and predict recurrence. Derived from extensive studies involving samples from healthy individuals and patients with renal and prostate cancer, these biomarkers have achieved remarkable sensitivity and specificity in both tissue and urine samples (Costa et al., 2010). This approach, being non-invasive, early, precise, and cost-effective, offers a viable option for the early detection of low-grade tumors, compared to traditional methods such as urine cytology and cystoscopy (Costa et al., 2010).

In colorectal carcinoma, the diagnostic landscape is still predominantly guided by the classic tumor-node-metastasis (TNM) methodology, especially in stage II where surgical interventions are common. However, these procedures carry a significant risk of tumor recurrence and fatal disease progression. To enhance accuracy and predictability, newer alternatives have been explored, involving specific biomarkers like carcinoembryonic antigen and carbohydrate antigen 19–9 (CA19-9), though the latter has faced criticism for its relative lack of specificity. Advances in technology such as microarray analysis, genomic screening, and sequencing have paved the way for more specific biomarkers. These biomarkers provide heightened precision in detecting microsatellite instability and microRNA instability (Luo et al., 2021). Biomarkers like KRAS, associated with viral oncogenesis, APC, linked to adenomatous polyposis coli, and TP53, coding for the tumor protein p53, have significantly contributed to the understanding of CRC pathogenesis and show promise in improving risk stratification and personalizing therapeutic approaches (Luo et al., 2021).

Finally, the epigenetic biomarker PHLPP2 has emerged as a significant indicator in pancreatic cancer. Research indicates that increased expression of PHLPP2 correlates with enhanced survival in patients with pancreatic adenocarcinoma. Studies have shown that vitamin C can elevate levels of 5-hydroxymethylcytosine in the promoter region of the PHLPP2 tumor suppressor gene, suggesting that VC-mediated DNA demethylation may positively regulate its expression. The epigenetic modulation of PHLPP2 could thus be pivotal in predicting recurrence and aiding in the treatment of pancreatic cancer (Chen et al., 2020).

In summary, the exploration of pharmacogenomic and epigenetic biomarkers in post-treatment evaluation opens new avenues for personalized medicine in cancer treatment, offering insights into patient prognosis, the efficacy of therapy, and potential future strategies in disease management based on the ethnicity (Paz-y-Miño et al., 2010; Paz-Y-Miño et al., 2015; López-Cortés et al., 2017b; 2020; López-Cortés et al., 2018; Yumiceba et al., 2020; Salas-Hernández et al., 2023).

### Optimizing pharmacoepigenetic interventions: the role of nutrition, inflammation control, and circadian rhythm regulation

Optimizing pharmacoepigenetic interventions requires a nuanced understanding of the roles played by nutrition, inflammation control, and circadian rhythm regulation (Masri et al., 2015; Barrero et al., 2022; Tan et al., 2022). Chronic inflammation is a well-documented root cause of various diseases, including a significant proportion (40%–60%) of carcinoma cases (Zitvogel et al., 2017). This inflammation can stem from multiple sources, such as dietary metabolic syndromes like high cholesterol (LDL), which are known to trigger chronic inflammation leading to the formation of atheroma plaques in blood vessels. Conversely, certain dietary components, like omega-3 fatty acids, have been shown to possess anti-inflammatory properties, highlighting the importance of diet in managing inflammation and its related epigenetic effects (Pisaniello et al., 2021).

Inflammatory dietary agents can exacerbate conditions in certain individuals. For instance, gluten can cause abdominal and/or colorectal inflammation in celiac patients, while lactose may lead to inflammation in the large intestine of lactose-intolerant individuals (Prodhan et al., 2022; Iversen and Sollid, 2023). Additionally, meat and its by-products, containing heme iron, aromatic amino acids, and LDL, contribute to chronic inflammation through the formation of the trimethylamine N-oxide byproduct and cytokine cascade activation (Janeiro et al., 2018).

Diet choices play a crucial role in modulating inflammation and, by extension, cancer risk (Soldati et al., 2018). For instance, plant-based diets, rich in anti-inflammatory agents and antioxidants, have been associated with a reduced risk of developing colorectal cancer and cardiovascular diseases. This association is attributed to their ability to mitigate inflammation and influence epigenetic markers related to disease progression, including high-sensitivity C-reactive protein (hsCRP), IL-18, interleukin-1 receptor antagonist (IL-1Ra), intercellular adhesion molecule 1 (ICAM-1), adiponectin, omentin-1, and resistin (Chovert, 2013; Menzel et al., 2020b). However, the specific impact of these diets on inflammatory biomarkers and epigenetic modifications requires further research to fully understand their potential in preventing disease and enhancing pharmacoepigenetic treatments (Menzel et al., 2020a).

Nutrition’s dual role as both a risk factor and a protective agent against epigenetic changes underscores its significance in disease outcomes. Nutrients such as folate (B9 or M) and polyphenols can influence the methylation of oncogenes and tumor suppressor genes, affecting cancer risk and progression (López-Cortés et al., 2013; 2015; 2017a; Bishop and Ferguson, 2015). Additionally, isothiocyanates in colorectal cancer demonstrate the ability to suppress DNA methyltransferase expression, acting as oncogene repressors and influencing cellular proliferation (Gupta et al., 2019). In breast cancer, they appear to reduce aggressiveness by making estrogen-receptor interactions more responsive to tumor proliferation inhibitors (Hernando-Requejo et al., 2019). This intricate interplay between diet, inflammation, and epigenetics is crucial for accurate disease prediction, diagnostics, and treatment.

Moreover, the circadian rhythm, often referred to as the body’s biological clock, plays a crucial role in regulating various cellular functions essential in cancer progression (Liu et al., 2023). It orchestrates key bodily activities such as sleep patterns, hormonal fluctuations, body temperature regulation, and metabolic processes (Pérez-Villa et al., 2023). Disruptions in this rhythm have been closely linked to an increased risk of developing cancer, a connection supported by epidemiological research (Kaakour et al., 2023). Studies have shown that disturbances in the circadian rhythm significantly raise the risk for various types of cancer, including breast, colon, prostate, and skin cancers (Zhou et al., 2022). Therefore, adopting healthy lifestyle habits, such as regular sleep routines, minimal nighttime light exposure, and a balanced diet, can significantly reduce cancer risk and support the effectiveness of pharmacoepigenetic interventions (Zhou et al., 2022).

In summary, the optimization of pharmacoepigenetic treatments necessitates a holistic approach that incorporates nutritional guidance, inflammation control, and circadian rhythm regulation. This approach not only promises to enhance the efficacy of treatments but also to prevent disease onset and progression through lifestyle modifications and dietary interventions.
